# Bioactive Loaded Novel Nano-Formulations for Targeted Drug Delivery and Their Therapeutic Potential

**DOI:** 10.3390/pharmaceutics14051091

**Published:** 2022-05-19

**Authors:** Sapna Kumari, Anju Goyal, Eda Sönmez Gürer, Evren Algın Yapar, Madhukar Garg, Meenakshi Sood, Rakesh K. Sindhu

**Affiliations:** 1 Chitkara College of Pharmacy, Chitkara University, Rajpura 140401, Punjab, India; ms.sapnakumari92@gmail.com (S.K.); anju.goyal@chitkara.edu.in (A.G.); madhukar.garg@chitkara.edu.in (M.G.); 2 Faculty of Pharmacy, Sivas Cumhuriyet University, 58140 Sivas, Turkey; edagurer@cumhuriyet.edu.tr (E.S.G.); evrenalginyapar@cumhuriyet.edu.tr (E.A.Y.); 3 Chitkara School of Health Sciences, Chitkara University, Rajpura 140401, Punjab, India; meenakshi.sood@chitkara.edu.in

**Keywords:** phytoconstituents, nano-formulations, liposomes, cubosomes, phytosomes, nanomedicines

## Abstract

Plant-based medicines have received a lot of attention in recent years. Such medicines have been employed to treat medical conditions since ancient times, and in those times only the observed symptoms were used to determine dose accuracy, dose efficacy, and therapy. Rather than novel formulations, the current research work on plant-based medicines has mostly concentrated on medicinal active phytoconstituents. In the past recent decades, however, researchers have made significant progress in developing “new drug delivery systems” (NDDS) to enhance therapeutic efficacy and reduce unwanted effects of bioactive compounds. Nanocapsules, polymer micelles, liposomes, nanogels, phytosomes, nano-emulsions, transferosomes, microspheres, ethosomes, injectable hydrogels, polymeric nanoparticles, dendrimers, and other innovative therapeutic formulations have all been created using bioactive compounds and plant extracts. The novel formulations can improve solubility, therapeutic efficacy, bioavailability, stability, tissue distribution, protection from physical and chemical damage, and prolonged and targeted administration, to name a few. The current study summarizes existing research and the development of new formulations, with a focus on herbal bioactive components.

## 1. Introduction

For this advanced and developed world afflicted with numerous health issues and ailments, nature has all the solutions. Nature offered various naturally existing bioactive plants for the treatment of various diseases, and such plants have been extensively used during the past several centuries all over the world due to their lesser side effects and extensive health benefits [1]. From ancient times, plants have been used for medicine and food [2]. The use of herbal medicine for basic health care in poor nations has undeniably grabbed the attention of the modern world [3]. Despite numerous advantages, pharmaceutical industries hesitate to finance natural product-based drug discovery and explore the synthetic compounds library for novel drug discovery. However, natural products and phytoconstituents have been extracted and screened for their benefits in primary health linked issues such as diabetes, cancer, microbial diseases, cardiovascular diseases, and inflammatory conditions because of their exclusive benefits, including lowered toxicity, low price, side effects, and outstanding health benefits [4].

Plant-based therapies have some limitations, such as poor lipid solubility, poor stability, and requiring a well-validated process for isolation and purification of constituent [5]. Indeed, this is the prime responsibility of the manufacturer to overcome these limitations and provide sufficient stability to the product and safer consumption by the patients. Typically, in traditional medicines, a limited amount of the drug has been reached at the target site. Most of the amount was dispersed all over the body based on the physicochemical properties of the medicine, resulting in lower therapeutic potency [6,7]. Herbal plants have a number of phytoconstituents that result in the instability of herbal formulation [8]. Delivery of the herbal formulations at the targeted site is a big challenge for most of the plant species that have medicinal significance. For instance, flavonoids, tannins, and terpenoids have water solubility, however they cannot cross the biological membrane, resulting in lesser absorption. Additionally, they possess a larger molecular size, resulting in diminished bioavailability and effectiveness [9].

To conquer these limitations, newer advanced drug delivery systems (DDS) have been developed for plant-based medicines. These include liposomes, phytosomes, ethosomes, transferosomes, nanostructured lipid carriers (NLCs), cubosomes, solid lipid nanoparticles (SLNs), hexosomes, microspheres, nanoparticles, and nano-emulsions [10]. These innovative DDSs have been associated with numerous improvements regarding targeted drug delivery, enhancement of solubility, stability, bioavailability, depletion of toxicity, and sustaining as well as controlling the release of the drug molecule [10,11].

Nanomedicine is an evolving area, using the application of nanoscience information and technology in remedial biology for treatment as well as disease prevention. It includes the use of nano-dimensional building resources such as nano-robots, diagnostic nano-sensors, sensory targets, and materials in living cells. An example includes the development of nanoparticle-based methods employed collectively for cancer diagnosis as well as cancer treatment [4]. In recent years, medicinal regulators authorized the first nanoparticle-based formulations that included lipid systems like liposomes and micelles [12]. Both of these formulations contained inorganic nanoparticles such as magnetic and gold nanoparticles [13]. The application of inorganic nanoparticles mainly emphasizes imaging, drug delivery, and medical activities. Additionally, nanostructures are reported to prevent drug deprivation in the intestinal tract and to facilitate the distribution of hydrophilic drugs over the targeted site. Additionally, nano-drugs resulted in improved drug bioavailability through the oral route, probably due to absorptive endocytosis mechanisms adopted by them. These nanostructures remained in the blood stream for a longer time and enabled drug release at a specified rate, resulting in reduced plasma fluctuation with reduced side effects. Because of their nano-size, these structures easily entered the cell membrane and facilitated drug uptake by cells, resulting in efficient target delivery. Additionally, the uptake of the nanosized structure by the cell is higher than larger particles, having sizes between 1 and 10 nm [14,15]. Therefore, these worked specifically for treating infected cells, resulting into higher efficacy and almost no adverse effects [4].

Concerning the nanomaterials utilizing drug delivery at a particular site, the choice of nano-based formulations depends on the physicochemical characteristics of drug molecules. Incorporating natural bioactives into nanoparticles using nanoscience is popular and rapidly growing in recent times. Most of the materials used are eco-friendly, biodegradable, bioadhesive, and of natural origin, which provides extensive benefits as well as distinctive size to the nano-formulations [16]. It offers various benefits for the treatment of cancer and many other ailments. Numerous extraordinary properties of natural compounds, including “tumor-suppressing autophagy” and antimicrobial properties, make them suitable for study in various life-threatening diseases. For instance, curcumin and caffeine exhibited autophagy [17], while antimicrobial and antibacterial effects have been demonstrated with cinnamaldehyde, curcumin, carvacrol, and eugenol [18,19]. The integration of drug molecules in nano-formulations resulted in enhanced oral bioavailability, identification, and control release of the drug. Considering an example, thymoquinone incorporated with nanocarriers showed a six-fold increase in oral bioavailability compared with free thymoquinone [20]. In addition, these nano-formulations also improved the pharmacokinetic properties of the natural bioactive, resulting in enhanced therapeutic effects [20]. Several reviews have been published on this tremendous technology, explaining its benefits to society. The current review is a compilation of the application of herbal-based nanoformulations developed through nanotechnology, which is one of the key novel drug delivery methods under investigation in recent years. These nanoformulations are thought to have a wide variety of benefits in comparison with conventional preparations of plant constituents, which include enhanced permeability, solubility, bioavailability, therapeutic activity, stability, improved distribution within tissues, and sustained delivery.

Nanotechnology is a fast-evolving branch of science due to its widespread application in other disciplines, making it more advanced and user-friendly. Nanotechnology is now used in nearly every major field of science, including agriculture, pharmaceutical sciences, medical sciences, computer sciences, food technologies, polymer sciences, textile technologies, chemical and biological sciences [21]. The pharmaceutical industry is experiencing a dilemma in drug research since they have strong therapeutic molecules, but their poorer water solubilities, low distribution, protein interaction, and short half-life lead to limited clinical usage. These nanotechnologies offer extensive benefits in this regard [22].

According to a report, the global market for nanotechnology-based products is expected to reach USD 91.8 billion by 2028. The Indian and Australian governments have committed around $20 million to create the “Australia-India Science Research Funding Program”. According to research released by BCC Research, the global value of the nanomedicines market in 2010 was 63.8 billion and 72.8 billion in 2011 [23].

Nanotechnology has numerous applications in many aspects of life, and it contributes significantly to the advancement of many scientific and industrial sectors, including information technology, energy, medical, national security, environmental science, food safety, and many more. Improved manufacturing methods, water purification systems, energy systems, physical enhancement, nanomedicine, better food production methods, nutrition, and large-scale infrastructure auto-fabrication are all major advantages of nanotechnology.

Changing the major properties of nanocarriers such as their constituents, sizes, shapes, and surface properties resulted in altered physio-chemical features of nano-formulations. The foremost aim of introducing nano-preparation is only to treat unwellness with supreme therapeutic potential and the least adverse effects. The use of an appropriate drug and nano-DDS-has been determined primarily by the biochemical and biophysical properties of the target drug [24]. Nevertheless, some hindrances such as toxicity could not be ignored while seeing their benefits. The lack of information about the toxicity and harmfulness of nanostructures is the main concern and undeniably needs more detailed studies to explore their maximum safety performance [25]. In view of the above facts, the current review article aimed to report various natural products based on nano-DDSs, significant use of natural nanomedicines in various ailments, along with various methods of preparations and their applications.

## 2. What Is a Nano?

**Figure 1:**
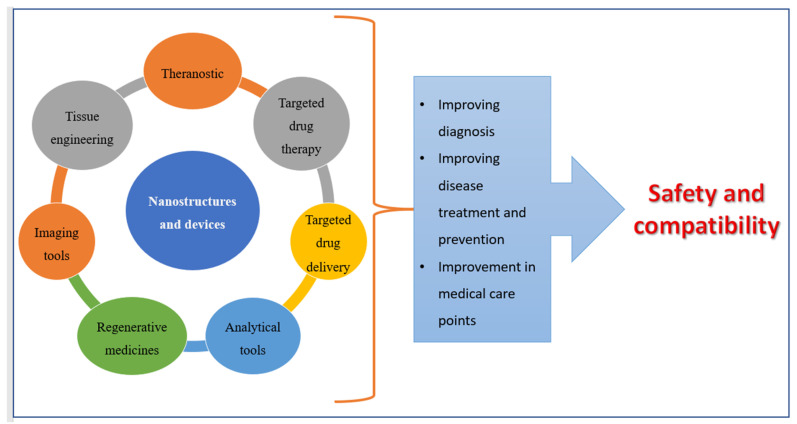
Applications of nanomedicines.

**Figure 2:**
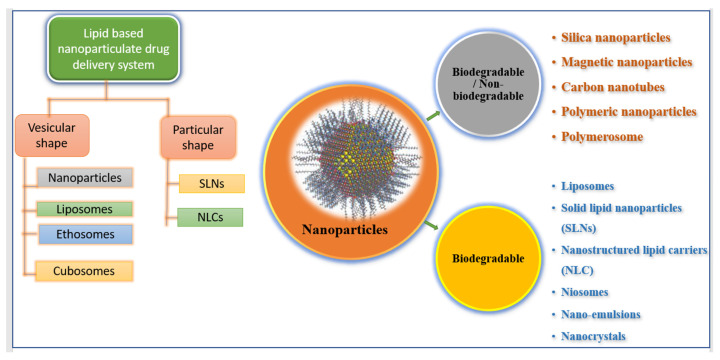
Illustrating various types of nano-formulations.

Advances in technology over the last two decades have resulted in the creation of nanoscale materials, which have resulted in a decrease in particle size and overall increases in surface area. Nanoparticles are particles with a size ranging from 1 nm to 1000 nm. The term “nano” is easily defined, but it encompasses a wide range of applications (Figure 1), including multiple nano-based systems made up of various types of materials used as nanocarriers (Figure 2) [26,27].

## 3. Nanotechnology-Based Drug Delivery Systems

### 3.1. Solid Lipid Nanoparticles (SLNs)

**Figure 3:**
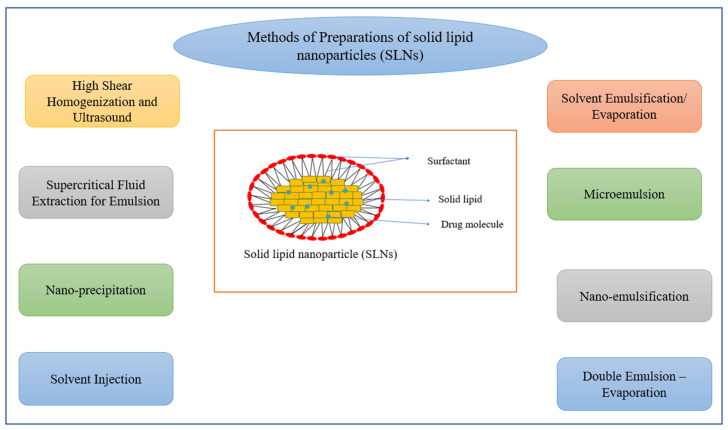
Methods of preparation of solid lipid nanoparticles (SLNs).

The SLN colloidal drug system was created in the early 1990s and has particle sizes ranging from 50 to 1000 nm. These are made of emulsifiers that help to keep a melted solid lipid dispersion water-stable [28]. For the preparation of SLNs (Figure 3), many procedures have been devised, the most prevalent of which are high-pressure homogenization (HPH) and micro-emulsification [29]. The main advantages of SLN include a lipophilic lipid matrix that allows pharmaceuticals to disperse, encapsulation of drug molecules such as medications, antigens, proteins, and nucleotides, and drug delivery to specified tissues and cells. Improved in vivo and in vitro drug stability, as well as reduced adverse effects, are among the unique characteristics of SLN [30]. SLN and nano-emulsions are quite similar, with the exception that SLN uses both solid and liquid lipids in their formulation, whereas nano-emulsions solely employ liquid lipids. In rats, the most often used SLN is puerarin-loaded SLN, which is characterized by quick absorption, increased bioavailability, and increased drug concentrations in targeted organs such as the brain and heart [31,32]. According to another study, “triptolide-loaded SLN” showed a significant reduction in myeloperoxidase (MPO) and glutathione (GSH) activities and acted as an anti-inflammatory and antioxidant product, resulting in improved solubility, reduced toxicity, and reduced irritation to the gastrointestinal tract (GIT), as well as avoiding higher local drug concentration and gradual drug release [33]. Table 1 has further examples.

### 3.2. Nanostructured Lipid Carriers (NLCs)

**Figure 4:**
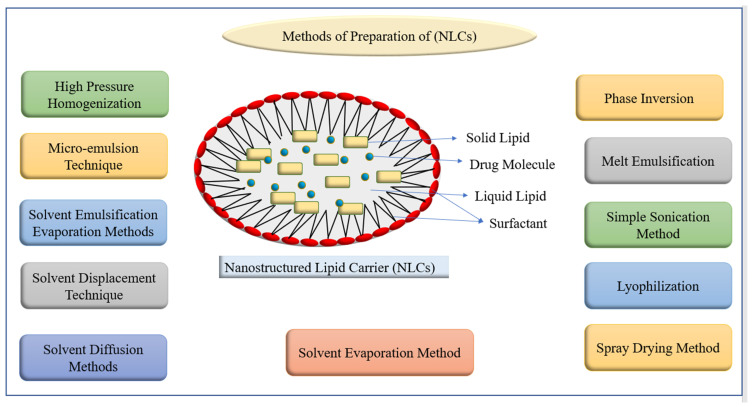
Methods of preparation of nanostructured lipid carriers (NLCs).

These are referred to as “second-generation lipid nanoparticles” because they are made up of a mixture of solid and liquid lipids and were produced from SLN with various lipid matrix flaws [42]. Solid lipids such as hydrogenated palm oil, glyceryl monostearate, stearic acid, and cetyl alcohol have been utilized in large quantities, while liquid lipids such as olive oil, mustard oil, castor oil, and cod liver oil have been employed. In this system, thiomersal has been utilized as a stabilizer [43]. NLCs outperform SLN because of their superior regulated drug release, enhanced drug loading ability, stability, and little drug loss during encapsulation [44]. Various studies focused on the entrapment of bioactives into NLC by altering water solubilities, controlling drug release, lengthening circulation time, co-delivery, routes of drug delivery, and enhancing gastrointestinal absorption and oral bioavailability. Different methods of NLCs preparation have been listed in Figure 4. NLCs carriers were found to be a better carrier for oral drug delivery, encapsulating various natural and synthetic bioactives. For example, tripterine, triptolide, and curcumin-loaded NLCs showed enhanced absorption that may be because of lipid components, smaller particle size, and surface contents. Silymarin-loaded NLC showed best examples, used clinically to overcome various liver diseases. Additionally, NLCs loaded with cardamom essential oil (CEO) have been developed successfully using food grade lipids olive oil and cocoa butter, showed small size and enhanced loading capacity (>25%), providing physical and chemical stability [45]. More examples of compound-loaded NLCs have been given in Table 2.

### 3.3. Nanocrystals

**Figure 5:**
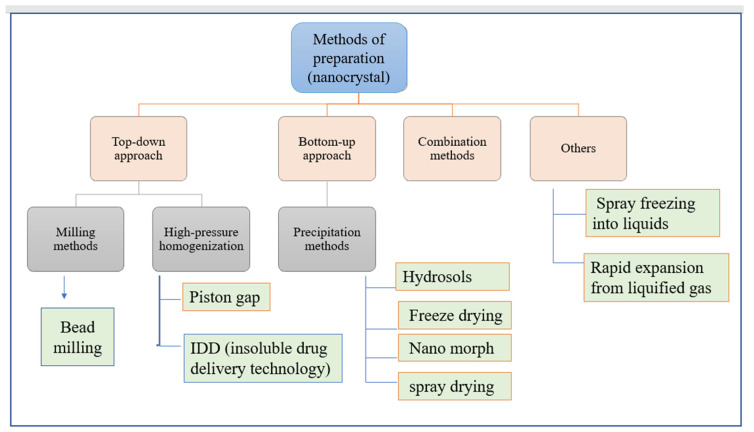
Methods of preparation of nanocrystal.

These are pure solid drug particle with sizes up to 1000 nm, primarily composed of 100% drug substance that is stabilized by stabilizer(s) or surfactant(s). Water, liquid polyethylene glycol (600), oil, or any “aqueous or non-aqueous” media has been used as a dispersing medium [60,61]. The noteworthy characteristics of nanocrystals enabled them to overwhelm difficulties such as increased “dissolution velocity, saturation solubility, and thickness to surface and cell membranes”. For the production of nanocrystals, two methodologies have been developed: a top-down approach and a bottom-up one. The top-down approach has defined approaches such as precipitation, high gravity-controlled precipitation technology, sono-crystallization, restricted impinging liquid jet precipitation technique, and multi-inlet vortex mixing techniques (Figure 5) [60]. In this process, the use of organic solvent, and its removal at the end, makes it moderately expensive. However, the bottom-up approach includes the application of high-pressure homogenization in grinding procedures [60]. Amongst all the methods, milling, precipitation, and high-pressure homogenization have been used most commonly for production. In nanocrystals, the mechanism followed by the drug for absorption includes solubility enhancement, suspension rate, and intestinal wall holding capacity [60]. These are associated with enormous advantages like enhanced solubility, disintegration, dissolution, bioavailability, and safer dosage form, and provide a higher level of safety because of their molecular size and surface properties [62]. Ni et al. developed a method by implanting “cinaciguat nano-crystals” into chitosan-based micro-particles, applied for hydrophobic drug delivery to the lungs. The polymer’s abilities of swelling and mucoadhesion enabled the continuous release of the drug, resulting in enhanced inhalation efficacy under diseased conditions [63]. More examples have been given in Table 3.

### 3.4. Nano-Emulsions

**Figure 6:**
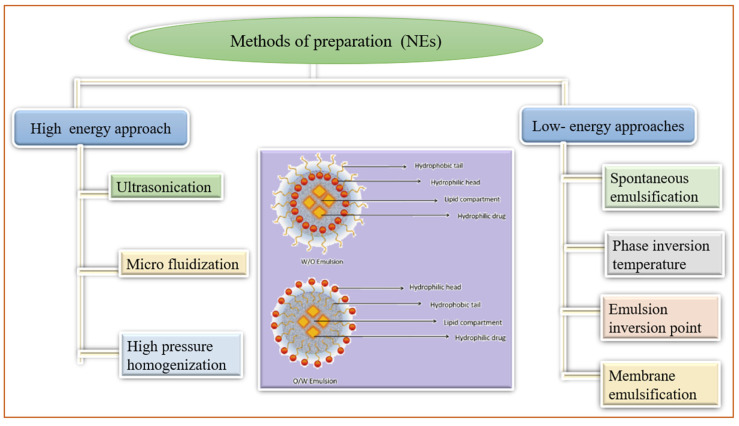
Methods of preparation of nano-emulsions (NEs).

Nano-emulsions (NE) are non-homogeneous, transparent colloidal dispersion systems of 100 nm size that are optically isotropic and thermodynamically stable. These are comprised of water and oil followed by the addition of co-surfactant and surfactant [71]. The lipophilic drug has been entrapped into the oil droplets, both in *o*/*w* and *w*/*o* suspensions. These oil droplets were engulfed by the macrophages and found in higher amounts in the liver, spleen, and kidneys. However, hydrophilic drugs were in the aqueous phase of *w*/*o* or *w*/*o*/*w* nano-emulsions. Figure 6 demonstrated different methods of NE preparation and the structure of nano-emulsions. Owing to their higher internal membrane permeability, these condensed to the lymphatic system, administered through intramuscular and subcutaneous routes [49]. The absorption of NE through the intestine has been attributed to lymphatic transport processes, resulting in amended oral bioavailability of the entrapped drugs [72]. The main characteristics of NE involve the stability of entrapped components, targeted sustained release, enhancing membrane permeability through the skin and mucous membranes, solubilizing components of different lipophilicities, improving drug absorption, lessening pain and allergy conditions, lowering viscosities, simple methods of production, and fewer chances of contamination [71,73]. The attractive properties of NE enabled their use as a vehicle for the distribution of essential oils, nucleic acid, drugs antimicrobial agents, repellents, and as an imaging agent [71,73]. In the past few eras, nano-emulsions have been amended for transdermal remedial use like phospholipids, Transcutol^®^P, fatty alcohol, alkyl poly-glycosides, and PEGylated fatty acid ester [74]. Numerous nano-emulsion formulations incorporating herbal drugs like camptothecin, genistein, rutin, oils of *Brucea javanica*, resveratrol, coixenolide, etc., with plenty of health benefits have been listed in the literature [75,76]. With great application scenarios of NE, more examples have been presented in Table 4.

### 3.5. Liposomes

**Figure 7:**
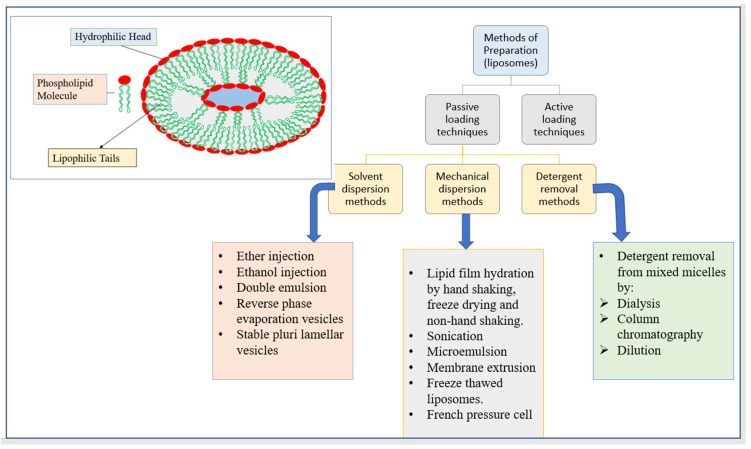
The structure of liposomes and different methods of preparation of liposomes.

Alec Bangham developed liposomes in 1960. These polar lipid nanoparticles are spherical in form and range in size from 50 to 450 nanometers. These can encase an “aqueous core” in “single or multiple lipid bilayers of natural or synthetic origin” into which it freely diffuses [89]. They have a membrane structure that is similar to that of cells. Liposomes are composed of materials that have both lipophilic and hydrophilic groups, allowing them to encapsulate both types of pharmacological molecules in the same structure [90]. Liposomes can increase drug solubility, drug delivery, the bioavailability of the entrapped drug, absorption of the drug within a cell, and drug distribution throughout the body both in vivo and in vitro due to their unique property of possessing phospholipid bilayers [91,92]. Figure 7 depicts the structure of liposomes as well as several liposome manufacturing methods.

The ADME profiles of drugs such as herbal, enzymes, and proteins can be modified accordingly, which is needed for preparing vaccines, nutraceuticals, and cosmetics. Additionally, some exclusive features like environmental protection of the entrapped drug molecule, devastating primary destruction of loaded bioactives, cost-effective, and quick treatment with least systemic morbidness, exaggerated their use in bio-medicine preparations [93].

Antibodies or ligands, on the other hand, can be added to liposomes to improve target specificity. Thangapazham et al., for example, developed curcumin-loaded liposomes coated with PSMA antibodies to treat the human prostate cancer cell lines LNCaP and C4-2B. As a consequence, improved targeted administration, 70–80% suppression of cell growth, and a 10-fold dosage advantage have been achieved [94]. Table 5 contains further instances of herbal compounds encapsulated in liposomes.

### 3.6. Phytosomes

**Figure 8:**
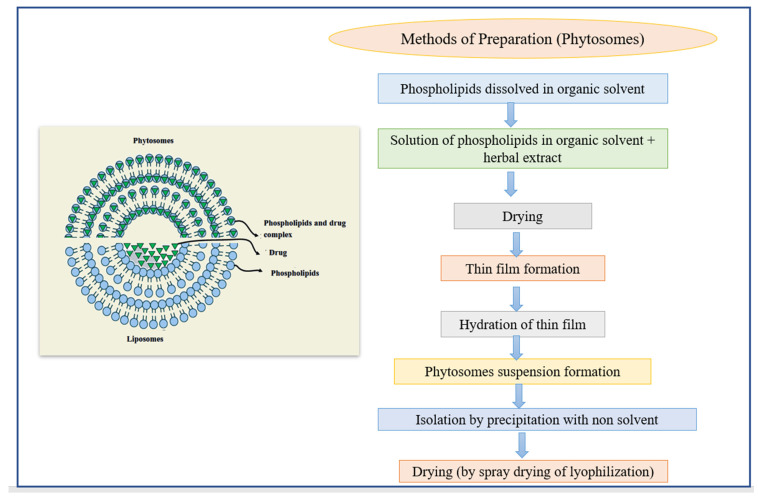
The structure of phytosomes and different methods of the preparation of liposomes.

Phytosomes are lipid-compatible molecular complexes that encapsulate pharmacological bioactive and water-soluble phytochemicals in phospholipids, resulting in increased absorption and bioavailability [111]. Hydrophilic phytochemicals, such as polyphenols and flavonoids, have lower absorption in the body due to their high molecular size, which made absorption across biological membranes difficult. These constraints have been overcome thanks to phytosome [71]. The uniqueness associated with them includes their molecular complex and chemical bond formation between plant material and phosphatidylcholine at a ratio of either 1:1 or 1:2 [112]. Structurally, phytosomes resemble liposomes, except for the entrapment of the material. In liposomes, the active material is dissolved in the medium present in the membrane layers, while in phytosomes the active material is a vital part of the membrane (Figure 8). The phytosomes created a better transition of the enterocyte cell membrane from a water-soluble to a lipid-soluble state, then inside the cell, reaching the bloodstream, and protecting entrapped herbal medications from stomach fluids and gut microorganisms. A large number of studies have been carried out to determine their use and qualities in comparison to other traditional delivery techniques. Recently, a group of researchers combined several flavonoids, including quercetin, kaempferol, and apigenin, into a single phytosome called flavonosome, which proved to be an effective antioxidant, hepatoprotective agent, and heat supplement [113]. In Table 6 below, we have included some more instances.

### 3.7. Ethosomes

Ethosomes are soft, non-invasive lipid-based elastic vehicles comprised of water, phospholipids such as phosphatidylcholine, phosphatidylethanolamine, phosphatidylserine, and phosphatidylglycerol, and about 30–45% ethanol and isopropyl alcohol [123,124]. The proportion composition of ethosomes improves their entrapment efficiency, topical drug delivery, and transdermal transport efficiency for both hydrophilic and lipophilic drugs. They provide delivery of ingredients into deeper tissue as well in blood circulation. Improved physical stability of ethosomes compared to liposomes is due to flexible lecithin bilayers [123]. On the contrary, ethosomes have some limitations like poorer stability, growing size from nanometer to micrometer caused by alcohol evaporation, and leakage of entrapped material after a while. Combining alcohol with trehalose and propylene glycol can help to overcome this weakness. To test their capacity as a transporter for delivering entrapped molecules to the skin in a rat model, “curcumin-encapsulated PEGlycated and conventional liposomes and ethosomes” were developed and tested. As a consequence, PEGlycated liposomes were shown to be the most promising ex vivo transdermal drug delivery technology, suppressing paw edema in the rat model to a greater extent [124]. More instances may be found in Table 7 (below).

### 3.8. Niosomes

**Figure 9:**
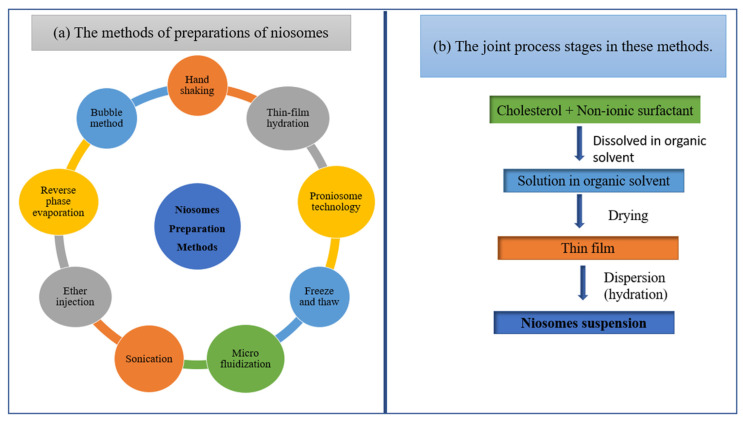
Schematic diagram of (**a**) the methods of preparations of niosomes and (**b**) the joint process stages in these methods.

These are nano-sphere vesicles of diameter ranging from 100 nm to 2 µm. These are non-ionic with a watery center, surrounded by non-ionic amphiphilic lipids in the lamellar phase [131]. Different methods of preparation include sonication, thin-film hydration, micro fluidization, multiple-membrane extrusion, remote loading, reverse-phase evaporation technique, and bubble method, as shown in Figure 9 [132]. The structure of niosome almost resembled the liposome, showing more stability, penetrating capability, and beneficial efficacy of the drug along with reduced toxicity [133]. The main advantages of niosomes are flexibility, cost-effectiveness, higher drug solubility and controlled release of the encapsulated bioactive, making them an effective peptide carrier and hemoglobin carrier, targeting vehicle for neoplasia, providing transdermal drug delivery of entrapped molecules. Niosomes, on the other hand, demonstrated extended drug circulation, skin retention, and penetration, as well as sustained drug release at the target location [134]. These are more stable nanocarriers than liposomes, with no notable toxicity, especially for topical usage in the treatment of skin problems such as skin cancer [135]. Table 8 shows several instances of niosomes that include natural remedies.

### 3.9. Cubosomes

These are viscous isotropic vesicles made up of mainly amphiphilic lipids (unsaturated monoglycerides) and thermodynamically stable surfactants such as poloxamers [141,142]. Due to properties like a large interior surface area per unit volume (approximately 400 m^2^/g) and a 3D structure with hydrophilic and hydrophobic domains, they easily entrap water-soluble and non-soluble, as well as amphiphilic, compounds. Its large interfacial surface can offer a variety of diffusion channels for the long-term release of entrapped drug molecules, and its lipid components are biodegradable, bio-adhesive, and digestible [143]. They are frequently created by dispersing or fragmenting the cubic phases of gel in the liquid phase.

**Figure 10:**
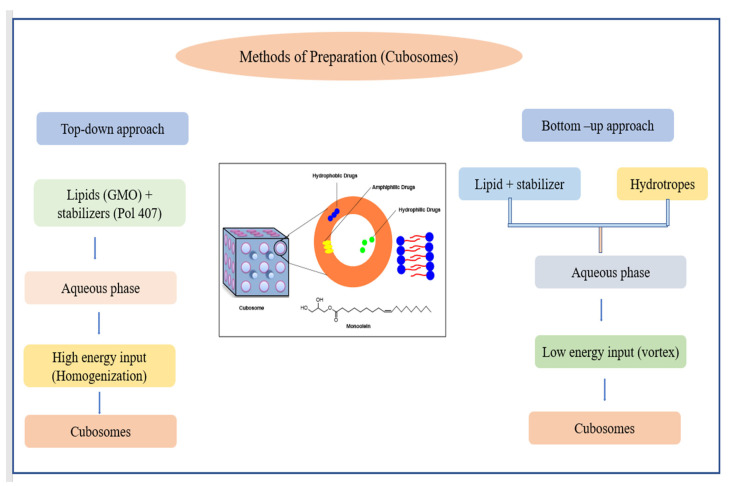
Top-down and bottom-up approaches for preparing cubosomes.

Two approaches, the top-down and bottom-up approaches, have been developed for cubosomes production (Figure 10). Somatostatin, indomethacin, insulin, rifampicin, etc., have been successfully encapsulated within the cubosomes. Moreover, peptides, anti-muscarinic effects, enzymatic effects, antibiotics, and analgesic administration are just a couple of small pharmacological uses of cubosomes that have been studied [144]. Because cubosomes have a structure that is almost identical to that of the stratum corneum, they may readily release the entrapped bioactive into the epidermis. Additionally, cubosomes’ features of adhesion and penetration increase imply their potential value in skin cancer (melanoma) treatment. Recently, a study was conducted to develop polymer-free cubosomes, for photodynamic treatment of the skin as well as bio-imaging of skin malignant tumors with extremely minimal cytotoxicity to the cutaneous system [145]. In Table 9, examples of cubosomes incorporating herbal bioactive have been listed.

## 4. Discussion

### 4.1. Nanotechnologies Applications

Nanotechnology has not only changed medicine but has also provided accuracy and precision for the treatment of many diseases. It has been considered an excellent technology for drug delivery as well as drug release at the target site. Nanotechnologies have been used for the applications such as fluorescent biological labels, detection of pathogens, drug and gene delivery, detection of proteins, probing of DNA structure and tissue engineering, etc. Moreover, tumor destruction through heating (hyperthermia), separation and purification of biological molecules and cells, MR imaging contrast enhancement, and phagokinetic studies are some others in the area of medicine for diagnosis and treatment of cancer. The nanotechnology provides advanced therapies with a reduced degree of invasiveness, and faster, smaller, and highly sensitive diagnostic tools which provide cost-effectiveness.

### 4.2. Drawbacks of Nanotechnology

The biocompatibility of nanoformulations is the major issue of concern. The ease with which nanotechnology-based treatment has been provided all over the world at basic levels (primary health care, government hospitals, etc.) and the cost of such treatments are the most crucial aspects. Moreover, the lack of knowledge about its toxicity and its impact on the biochemical pathways, human body, and environment needs to be studied very closely. Society’s ethical use of nanomedicine and the concerned safety issues pose a serious question to the researchers [151,152,153,154,155,156,157].

### 4.3. Ethical Concern

Nanoscience and nanotechnology, like any new scientific approach, are involved in a dispute regarding the degree of usefulness. Research on the ethical, legal, social, and environmental aspects of nanoscience and nanotechnology has been recognized as a viable subject of investigation in Western countries. Because nanomedicine is a relatively new field of science and nano-technology-based drug treatments differ significantly from existing treatments, there may be considerable uncertainty and difficulty in regulating the nanotechnology-based treatment and its applications. As a result, it may be difficult to regulate nanotechnology-based treatment and applications [158].

## 5. Conclusions

For the last few decades, nanocarrier-based DDSs have been investigated as a new drug transporter because of the benefits offered by the active ingredients. Naturally occurring medicines contained a wide range of therapeutic characteristics that should be investigated using advanced drug delivery methods. Poorer water solubilities and bioavailability are some limiting issues associated with these methods. Researchers developed new methods either by entrapping into a drug carrier or by modulating drug structure by adding some stable groups. The primary element to consider while developing any formulation is the necessity of developed formulation to cross biological membranes. The main criteria for this are lipid solubilities and molecular size of the drug. Recent studies have predicted their applicability in the treatment of diseases such as diabetes, cancer, anemia, hypertension, and a variety of others, and current research has attempted to address current challenges by applying nanocarriers methodologies. Nanocarriers created a low drug level in the blood, resulting in reduced toxicity, which is advantageous for patients who required medications on a daily basis. The developments in nanomedicine achieved to date have changed the techniques for the administration of drugs in our body, even though the underlying mechanism, safety, and toxicity profile of nanomedicine is still being developed. The technology developed to identify illnesses and even combined therapy and diagnosis a reality is realistic thanks to current developments in nanomedicine.

In conclusion, pharmaceutical nanotechnology is an emerging field of science in every aspect of maintaining the drug stability, solubility, absorption, and bioavailability of poorly water-soluble and less bioavailable drugs. Additionally, nanotechnology-based systems enhance the targeted delivery and sustained delivery of the entrapped material, leading to efficient therapeutic potency with reduced side effects. In numerous laboratories in India, pharmaceutical development of nanotechnology-based DDSs is being undertaken with zeal. These are being studied in vitro for release patterns and in vivo in animals for pharmacokinetics, but not often for efficacy. There is a lack of information on clinical research and the development of nanotechnology-based DDSs utility in patients. It is required to involve any pharmacologists in the investigation of pharmacokinetics and pharmacodynamics of DDS to know if the products have reached their meaningful outcome—clinical use.

## Figures and Tables

**Table 1:** SLN encapsulating natural bioactive.

SLNs Loaded with Natural Bioactive	Plant Source	Limitations of Free Drugs	Advantages of Loaded Drug Molecules	References
Triptolide incorporated SLN	*Tripterygium wilfordii* Hook F	Poor water solubility and high toxicity,	Improved solubility, hyperemia, reduced toxicity, irritation to GIT, etc.	[33]
Puerarin-loaded SLN	*Pueraria lobata* (wild) *Howe*	Poor water solubility and low oral bioavailability	3-folds increase in absorption and bioavailability improved tissue concentration in targeted organs (heart and brain)	[31,32]
Noscapine PEG conjugated SLN	Papaveraceae family	Shorter half-life, less efficacy to glioblastoma cells	Improved biological half-life, and anticancer efficacy in glioblastoma in vitro and in Swiss male albino mice induced with brain cancer.	[34]
Tetrandrine-loaded SLN	*Stephania tetrandra* S. Moore	Lesser bioavailability and drug release	Improved bioavailability, in vitro drug release, cellular uptake into human lens epithelial cell line (SRA 01/04)	[35]
Cantharidin-loaded SLN	*Mylabris phalerata pallas or mylabris cicchorii linnaeus*	Lesser bioavailability and drug release	Sustained drug release without a burst effect, improved bioavailability when administered orally in rats induced with gastric mucus membrane irritation.	[36]
Hydroxycitric acid-loaded SLN	*Garcinia cambogia*	Low bioavailability	Increased bioavailability tested on Wistar rat, anti-obesity medication	[37]
*Ginkgo biloba* leaf extract-loaded SLN	*Ginkgo biloba*	Low bioavailability	Improved oral bioavailability at a 5 mg/kg dose, causing blood coagulation at higher doses i.e., 50 mg/kg.	[38]
*Aloe vera*-loaded SLNs	*Aloe vera*	Cause irritation to the skin on multiple uses in some cases	Incorporated into sunscreen cream, SPF was found to be as per the marketed formulation.	[39]
*Zataria multiflora* essential oil (ZMEO) containing SLN	*Zataria multiflora*	Mosquito repellant properties at higher doses	Improved mosquito repellent activities, three times increase in protection time of nano-formulation compared to non-formulated essential oil	[40]
Witepsol-loaded SLNs	Cocoa butter	Lower stability	Suitable vehicle for herbal extracts, higher stability, and proper release profile in the intestine.	[41]

**Table 2:** NLCs incorporated bioactives.

Drug-Loaded NLCs	Plant Source	Limitations of Free Drugs	Advantages of Loaded Drug Molecules over Conventional Systems	Reference
Cardamom essential oil-loaded NLCs	*Elettaria cardamom*	Low antimicrobial activities	Protect antimicrobial activity of the plant extract, used as food supplement	[46]
Thymoquinone-loaded NLCs	*Nigella sativa*	Low bioavailability	Enhanced bioavailability and oral drug delivery, antioxidant potential, improved liver biomarkers affected with PCM induced hepatotoxicity	[47]
Citral-loaded NLCs	*Cymbopogon citratus*	Low solubility	Improved water solubility and sustained drug release	[48]
β-Elemene incorporated NLCs	*Nigella damascena* L.	Low bioavailability and anticancer efficacy	Improved bioavailability in male wistar rats and anti-tumor efficacy in H22 hepatoma induced in Kunming mice, reduced venous irritation after i.v., injection in New Zealand white rabbits.	[49]
Zerumbone-loaded NLCs	*Zingiber zerumbet* L. Smith	Low solubility	Improved water solubility, bioavailability, and sustained drug release with enhanced anticancer activities both in vitro and in vivo.	[50,51]
Baicalin-loaded NLCs	*Scutellaria baicalensis*	Low solubility and bioavailability	Improved sustained drug release and antidiabetic effect of baicalin	[52]
Berberine incorporated NLCs	*Coptis chinensis*	Low bioavailability	Enhanced anti-inflammatory potential of the berberine, improved ulcerative colitis symptoms.	[53]
Curcumin-loaded NLCs	*Curcuma longa*	Low solubility and bioavailability	Improving impressions of DR5 proteins, enhanced caspase 8 and caspase 3 activities, enhanced apoptosis in hepatocellular carcinoma	[54]
Hesperidin and clarithromycin-loaded NLCs	*Flavanone glycoside*	Low bioavailability	Improved sustained and controlled drug release that can be used to increase the rate of *H. pylori* eradication.	[55]
Diosgenin and *Glycyrrhiza glabra* extract-loaded NLCs	*Dioscorea deltoidea* *Glycyrrhiza glabra*	Possessed lessened anti-inflammatory properties	Inhibition of pro-inflammatory cytokines, TNF-α, IL, and enhanced anti-inflammatory properties	[56]
Cinnamaldehyde-loaded (NLC)	*Cinnamomum ceylanicum*	Low bioavailability and shelf life	Total bacteria and fungi count in the treated CA-loaded NLC samples was about 3.5 log CFU/g less than the control. CA-loaded NLC can extend the shelf life of date fruit without any undesirable impacts on sensory attributes.	[57]
Ursolic acid-loaded NLCs	*Pentacyclic terpene acid*	Low solubility	Animals infected with *Leishmania (Leishmania) infantum* and treated with UA-NLC showed lower parasitism than the infected controls, Increased protective immune response, spleen and liver preservation, and the normalization of hepatic and renal functions.	[58]
Naringenin (NGN) incorporated NLCs	*Citrus fruits and tomato*	Poor water solubility	Elevated drug release rate in simulated intestinal solutions in vitro, improved transepithelial transport in MDCK cells, improved oral absorption in mice, enhanced inhibitory effects of NGN on MCD diet-induced mouse NAFLD.	[59]

**Table 3:** Nanocrystals encapsulating herbal medicines.

Nanocrystals of Herbal Compounds	Plant Source	Limitations of Free Drugs	Results and Outcomes of Loaded Formulations	References
Rutin incorporated nanocrystals (RNs)	Buckwheat, eucalyptus	Poor water solubility	Improved water solubility and bioavailability, RNs showed 100 times more cytotoxic effect on HN5 cells, decreased expressions of *Bcl-2 mRNA*	[64]
Cellulose nanocrystals isolated from Amla pomace	*Phyllanthus emblica*	Free drugs do not possess this property	Cellulose nanocrystals help in converting food industry waste into valuable products, and act as a low-cost precursor for various nanoformulations	[65]
Curcumin (CUR) and beclomethasone dipropionate (BDP) nanocrystals	*Curcuma longa*	Poor water solubility and bioavailability	Improved water solubility and bioavailability, therapeutic efficacy, improved lung delivery of active molecule, improved asthmatic conditions	[66]
Silymarin nanocrystals	*Silybum Marianum*	Low solubility	Improved drug dissolution profile, sustained drug release	[67]
Ethanol extract from *Ficus glomerata* nanocrystals	*Ficus glomerata*	Lesser biological properties	Showed comparable activities against *Aedes aegypti*, *Culex quinquefasciatus*, and *Anopheles stephensi* to the conventional neem oil-based nano-emulsion and repellent properties are more effective than commercial formulation.	[68]
Puerarin	*Pueraria lobata*	Low bioavailability	Enhanced oral bioavailability and upgraded brain accumulation for the treatment of Parkinson’s disease (PD)	[69]
Resveratrol nanocrystals	Natural polyphenol	Low water solubility	Improved water solubility and dermal patches preparation for treatments of acne and skin diseases	[70]

**Table 4:** Nano-emulsions containing herbal bioactive.

Herbal Nano-Emulsion	Plant Source	Limitations of Free Drugs	Results and Outcomes of Loaded Bioactive	References
Hydroxy-safflor yellow A NE	*Carthamus tinctorius*	Low absorption and bioavailability	Enhanced systemic absorption and improved bioavailability.	[77]
Oregano oil NE	*Origanum vulgare*	Limited spectrum antibiotics	Reduced and controlled growth of food-borne bacteria (*L. monocytogenes, S. Typhimurium,* and *E. coli*) on fresh lettuce.	[71]
Elemene oil NE	*Curcuma* species	Low stability and bioavailability	Improved stability and oral bioavailability in Sprague Dawley rats than a commercial elemene emulsion.	[78]
Quercetin NE	Many plant parts like nuts	Low skin penetration cause skin irritation	Increased cutaneous permeability reached the systemic circulation with lower skin retention.	[79]
Basil oil NE	*Ocimum basilicum*	Have lesser antibacterial activity	Antibacterial activity against pure *E. coli* culture	[80]
*Nigella sativa* L. NE	*Nigella sativa* L.	Limited free radicle scavenging activity	Enhanced and dose-dependent radical scavenging capacity in the DPPH assay (IC50 of about 47 µg/mL), reduced bioavailability of A2780 cancerous cells, NE showed pro-apoptotic, antioxidant, and anticancer effects.	[81]
Linseed oil NE	*Linum usitatissimum* seed	Poorer stability and penetration through the skin membrane	Improved stability and physicochemical properties for topical applications, suitable for atopic dermatitis evaluated through in vitro and in silico studies.	[82]
Cumin tincture-loaded NE	*Cuminum cyminum* L.	Limited free radicle scavenging activity and antibacterial properties	Good and dose-dependent radical scavenging capacity, antioxidant, anti-angiogenic effect, antibacterial activity against *S. aureus* and *K. pneumonia*.	[83]
Essential oil NE	*Alhagi maurorum*	Limited bioavailability	Enhanced antibacterial and antibiofilm activity, identified as antimicrobial agents against antibiotic-resistant bacteria.	[84]
*Nelumbo nucifera* crude extracts.	*Nelumbo nucifera*	Poorer stability	Enhanced stability and antimicrobial activities act as an alternative active ingredient for skin bacterial infection.	[85]
Peppermint and rosemary essential oils NE	*Mentha piperita,* Mint family Lamiaceae	Dermal irritation and toxicity	Reduced osteoarthritis pain via increasing antioxidant capacity and improving the histopathological features of the rats’ knee joint.	[86]
Essential oil NE	*Thymus vulgaris*	Limited antifungal properties	Obtained as promising alternatives for the treatment of cutaneous mycoses, especially when the etiological agents are resistant to conventional antifungal drugs.	[87]
Essential oil NE	*Myristica fragrans or Lavandula dentata*	Poorer stability	Improved physical and chemical stability in different temperature and storage conditions	[88]

**Table 5:** Liposomes containing herbal bioactives.

Liposomes of Herbal Compounds	Plant Source	Limitations of Free Drugs	Results	Reference
Baicalin-loaded liposomes	Root of *Scutellaria baicalensis* Georgi)	Low water solubility and drug release	Improved solubility, sustained release, enhanced drug concentration in brain tissue after i.v. administration in rats	[95]
Polydatin-loaded liposomes	Root and rhizome of *Polygonum cuspidatum* Sieb	Poorer solubility and bioavailability	Enhanced oral bioavailability, improved solubility, and sustained release in vitro.	[96]
Paclitaxel-loaded/PEGylated/saturated PC-based liposomes	The bark of *Taxus brevifolia* or pacific yew	Low solubility and bioavailability	Improved bioavailability, solubility, biodistribution, and intracellular uptake.	[97,98]
Naringenin-loaded liposomes	Immature orange fruit and the peels of grapefruits)	Poorer solubility and bioavailability	Improved stability, solubility, bioavailability, and tissue distribution the sustained release both in vivo and in vitro after oral administration.	[99]
Sterols-loaded liposomes	*Flammulina velutipes*	Limited solubility and bioavailability	Improved water solubility, oral bioavailability, and tissue distribution in liver tumor-bearing Kunming mice.	[100]
Quercetin-loaded liposomes	Flavonoids	Reduced solubility and bioavailability	Improved water solubility, and oral bioavailability, used in wound healing	[101]
Curcumin-loaded liposomes	*Curcuma longa*	Low anticancer properties	Anticancer and anti-inflammatory potential	[102]
Curcumin-loaded thiolated polymer-coated liposomes	*Curcuma longa*	Low bioavailability	The improved therapeutic index of curcumin, Aphthous ulcer	[103]
Colchicine-loaded liposomes	*Colchicum autumnal, gloriosa superba* extract	Poorer drug release	The anti-gout drug, improved drug transport	[104]
Liposomal neem gel	*Azadirachta indica* leaves	Limited antibacterial spectrum	Enhanced anti-bacterial activities	[105]
Capsaicin liposomes	Genus *capsicum*	Low bioavailability	Enhanced bioavailability, treating neuropathic pain	[106]
Brucine liposomes	*Nux vomica*	Low bioavailability and showed side effects	Reduced side effects of brucine like violent seizures	[107]
Guggul liposomes	*Commiphora Mukul.*	Low bioavailability	Improved anti-inflammatory properties.	[108]
*Asparagus racemosus* liposomes	*Asparagus racemosus*	Low bioavailability	Improved anti-inflammatory properties.	[109]
*Polygonum aviculare* L. *herba* (PAH) extract entrapped liposomes quercetin-entrapped liposomes	*Polygonum aviculare* L.Quercetin	Low cell viability	Moderately efficient on cell viability while quercetin-loaded liposomes showed increased cell viability and provide better endothelial protection compared to free quercetin and PAH-loaded liposomes	[110]

**Table 6:** Phytosomes containing herbal medicines.

Phytosome	Plant Source	Limitations of Free Drugs	Results and Outcomes	Reference
Epigallocatechin gallate-loaded phytosome	*Camellia sinensis*	Low stability and bioavailability	Improved solubility and bioavailability. Physicochemical stability through organoleptic, water content, and physicochemical properties at various temperatures	[114]
Rutin-loaded phytosome	Citrus fruits	Low stability and poor drug release	Improved solubility, stability, releasing dynamics and bioavailability in vitro, good antioxidant agent	[115]
Soybean seed Phytosome-based thermogel	*Glycine max* L.	Low drug absorption and solubility	Improved absorption, instability, insolubility, and fast releasing. A clear reduction in body weight, adipose tissue weight, studied in vivo.	[116]
Gingerol-loaded phytosome	*Zingiber officinale*	Poor stability and drug absorption	Improved stability, oral absorption, bioavailability, sustained release, showing potent antioxidant, antibacterial (against *Staphylococcus aureus* and *E. coli*), and anti-inflammatory activities in vitro.	[117]
*Butea monosperma* flower extract-loaded phytosome	*Butea monosperma*	Poor water solubility and bioavailability	Improved solubility, bioavailability, stability, and release dissolution pattern and showed significant free radical scavenging activity in vitro using the DPPH model.	[118]
*Swertia perennis* L.-loaded phytosome	*Swertia perennis* L.	Poor drug release profile.	Improved entrapment efficiency and in vitro drug release of embedded phytomedicine.	[119]
Aloe Vera extract-loaded phytosome	Aloe Vera	Limited anticancer activity	Inhibitory effect on the growth of the MCF-7 cancer cell line, enhanced oral delivery of aloe vera, making its use in cancer therapy.	[120]
*Morinda lucida* extract-loaded phytosome	*Morinda lucida*	Limited antimicrobial activities	In vivo, anti-plasmodium studies confirmed a higher anti-malarial effect comparable/similar to the standard drug (artesunate).	[121]
Aqueous extract of stem bark and lecithin of *Tecomella undulata*-loaded phytosome	*Tecomella undulata*	Poor drug release profile and bioavailability	Good entrapment efficiency and drug release in nano sizes (up to 90%), improved bioavailability without resorting to any pharmacological adjuvant or structural modification of the ingredients.	[122]

**Table 7:** Ethosomes incorporated with herbal medicines.

Herbal Drug-Loaded Ethosomes	Plant Source	Limitations of Free Drugs	Results and Outcomes	References
Apigenin-loaded ethosomes	From many fruits and vegetables such as chamomile	Low bioavailability	The strong anti-inflammatory activity caused by ultraviolet B light exposure after topical application	[125]
*Berberis aristata* extract-loaded ethosomal gel	*Berberis aristata*	Lesser drug penetration and bioavailability	Enhanced permeation profile and transdermal delivery of the extract provide a better approach for dermatological disorders	[126]
Cryptotanshinone-loaded ethosomal gel	*Salvia miltiorrhiza*	Lesser drug penetration and bioavailability	Enhanced transdermal flux, skin permeation, and deposition on pigskin in vitro. Improved anti-acne activity with reduced skin irritation in the ear of rabbit model associated with ethosomal gel.	[127]
Colchicine trans ethosomal gel	From dried corns and seeds of plants of the genus *Colchicum*	Poor stability, solubility drug release bioavailability	Improved stability, solubility, sustained release, bioavailability, and skin diffusion in vitro.Enhanced drug accretion, tissue biodistribution, and skin permeation in an ex vivo using Sprague Dawley rats’ back skin	[123]
Piperine-loaded ethosomes	*Piper nigrum*	Lesser drug penetration and bioavailability	Ethosomal cream showed higher deposition in skin layers, non-toxic to HaCat cell lines, and novel drug carrier for management of atopic dermatitis.	[128]
*Achillea millefolium* L.-loaded ethosomes	*Achillea millefolium* L.	Limited free radical scavenging activities and drug release	Enhanced free radical scavenging activities by about 88%, improved drug release by about 79.8%	[129]
*Sambucus nigra* L. Extract-loaded ethosomes	*Sambucus nigra* L.	Cause skin irritation	Possessed collagenase inhibition activity, excellent skin compatibility, recognized as a potent cosmeceutical ingredient	[130]

**Table 8:** Niosomes loaded with herbal bioactive.

Herbal Medicine-Loaded Niosomes	Plant Source	Limitations of Free Drugs	Results and Outcomes	References
*Permacoce hispida*-loaded niosome	*Permacoce hispida-*	Poor stability and bioavailability	Improved stability, bioavailability, sustained release, and permeability in vitro. Enhanced anti-tuberculosis in vitro.	[136]
Embelin-loaded niosome	*Embelia ribes Burm.*	Poor stability and bioavailability	Improved stability, bioavailability, sustained release, and biocompatibility in vitro. Upgraded streptozotocin-induced diabetes in Albino Wistar rats with potential antioxidant activity.	[137]
Lawsone-loaded niosome	Persian Henna, *Lawsonia inermis*	Poor stability and bioavailability	Improved stability, bioavailability, sustained release, and in vitro permeability. Significantly improved the antitumor activity in MCF-7 cells in vitro.	[138]
Rosemarinic acid-loaded niosome	*Rosmarinus officinalis*	Limited drug release and drug stability	Improved sustained delivery of Niosomal gel of rosmarinic acid to bacteria (*Propionibacterium acne* and *Staphylococcus aureus*) infected cells in vitro (anti-acne vulgaris). Improved delivery of naturally occurring antimicrobial and anti-inflammatory agents, in deeper tissues of skin in vivo using Swiss albino mice.	[139]
*Nerium oleander* -loaded niosome	*Nerium oleander*	Limited antioxidant activity and bioavailability	Improved cell effectiveness and tolerability of active substances. Improved in vitro cytotoxicity toward cervical and alveolar cancer cells (HeLa and A549) using MTT assay. Displayed potential antioxidant activity in vitro using DPPH radical scavenging assay.	[140]

**Table 9:** List of herbal bioactive-loaded cubosomes.

Herbal Medicine-Loaded Cubosomes	Plant Sources	Limitations of Free Drugs	Results and Outcomes	References
Piperine-loaded cubosomes	Fruits of the piperaceae family	Low stability	Improved stability, hydrophobicity, the enhanced and cognitive effect of piperine, displayed anti-inflammatory, anti-apoptotic, and antioxidant effects.	[146]
Curcumin-loaded cubosomes	*Curcuma longa* L.	Low stability	Upgraded stability, production of nanosized vesicles, and enhanced anti-bacterial properties in topical drug delivery.	[147]
Achyranthes bidentata-loaded cubosomes	Polysaccharides	Low stability and immunomodulatory effect	Improved stability, immunomodulatory effect, and displayed fewer toxicities to splenic lymphocytes in vitro.	[148]
Capsaicin incorporated cubosomes	All plants of the capsicum family	Cause skin irritation	Lowered skin irritation, enhanced stability under light and heat, sustained delivery for transdermal administration of capsaicin.	[149]
Essential oil of *Citrus trifoliata* L. incorporated cubosomes	*Citrus trifoliata* L.	Limited insecticidal activities	Enhanced insecticidal and fungicidal activities against Fusarium oxysporum, Spodoptera littoralis, and Fusarium solani.	[150]

## Data Availability

The data supporting the findings of this study are available within the article.
